# Public perceptions and attitudes toward thalassaemia: Influencing factors in a multi-racial population

**DOI:** 10.1186/1471-2458-11-193

**Published:** 2011-03-30

**Authors:** Li Ping Wong, Elizabeth George, Jin-Ai Mary Anne Tan

**Affiliations:** 1 Department of Social and Preventive Medicine Faculty of Medicine, University of Malaya, Kuala Lumpur, Malaysia; 2 Julius Centre, University of Malaya, Kuala Lumpur, Malaysia; 3 Centre for Population Health, University of Malaya, Kuala Lumpur, Malaysia; 4 Hematology Unit, Department of Pathology, Faculty of Medicine and Health Sciences, Universiti Putra Malaysia (UPM), Serdang, Selangor, Malaysia; 5 Department of Molecular Medicine, Faculty of Medicine, University of Malaya, 50603, Kuala Lumpur, Malaysia

**Keywords:** thalassaemia, public awareness, attitudes, nationwide survey, multi-racial population

## Abstract

**Background:**

Thalassaemia is a common public health problem in Malaysia and about 4.5 to 6% of the Malays and Chinese are carriers of this genetic disorder. The major forms of thalassaemia result in death *in utero *of affected foetuses (α-thalassaemia) or life-long blood transfusions for survival in β-thalassaemia. This study, the first nationwide population based survey of thalassaemia in Malaysia, aimed to determine differences in public awareness, perceptions and attitudes toward thalassaemia in the multi-racial population in Malaysia.

**Methods:**

A cross-sectional computer-assisted telephone interview survey of a representative sample of multi-racial Malaysians aged 18 years and above was conducted between July and December 2009.

**Results:**

Of a total of 3723 responding households, 2846 (76.4%) have heard of thalassaemia. Mean knowledge score was 11.85 (SD ± 4.03), out of a maximum of 21, with higher scores indicating better knowledge. Statistically significant differences (*P *< 0.05) in total knowledge score by age groups, education attainment, employment status, and average household income were observed. Although the majority expressed very positive attitudes toward screening for thalassaemia, only 13.6% of married participants interviewed have been screened for thalassaemia. The majority (63.4%) were unsupportive of selective termination of foetuses diagnosed with thalassaemia major.

**Conclusion:**

Study shows that carrier and premarital screening programs for thalassaemia may be more effective and culturally acceptable in the reduction of pregnancies with thalassaemia major. The findings provide insights into culturally congruent educational interventions to reach out diverse socio-demographic and ethnic communities to increase knowledge and cultivate positive attitudes toward prevention of thalassaemia.

## Background

Thalassaemia is one of the most common genetic blood disorders in the world. There are approximately 240 million people worldwide who are heterozygous for β-thalessemia and approximately 200,000 affected homozygotes are born annually [1]. Like many other countries, thalassaemia also pose an important public health problem in Malaysia. An approximately 4.5% of Malaysians are carriers of β-thalessemia, and the affected births annually are estimated at 2.1 per 1,000 with an estimated 5,600 patients with transfusion dependent β-thalessemia in Malaysia [2]. The Malaysian Chinese posses the Southeast Asian deletion that causes Bart's hydrops foetalis, a fatal condition in α-thalassaemia [3].

Malaysia is a fast developing country in Southeast Asia with a population of 27.7 million, which encompasses a majority of Malay (50.8%) and other ethnic groups, mainly Chinese (23.0%), indigenous peoples of Sabah and Sarawak (11.0%), Indian (6.9%), and other minority groups making up the remaining 8.3% of the population [4]. The Malays are the original inhabitants of Peninsula Malaysia and together with the indigenous peoples of Sabah and Sarawak, are collectively called "Bumiputeras" (sons of the soil). The Malaysian Chinese were mainly from the southeastern China provinces of Zhejiang, Fujian, Guangdong, Hainan and Guanxi. Malaysian Indians are a group largely descended from those who migrated from southern India. The Malays are predominantly Muslim, the Chinese are largely followers of Buddhism, Taoism, and Confucianism, and the majority of the Indian Malaysians are Hindus or Sikhs. Thalassaemia is more prominent among the Malays and Chinese, whereas the Indians form only a small percentage of those with thalassaemia. [5].

Management of patients with thalassaemia constitutes a heavy burden for affected families and the health care system. Moreover, social stigma associated with having thalassaemia have significant psychosocial and emotional impact on patients and their families. In India, being a thalassaemia carrier caused social isolation, marital tensions and stigmatization [6,7]. Prevention of the birth of children with thalassaemia major is, therefore, important to reduce the prevalence of this disorder. Empirical evidence indicates that prenatal diagnosis has dramatically reduced the disease burden [1,8,9]. Nevertheless, lack of knowledge and awareness about the disorder, its consequences, and psychosocial and cultural issues may serve as barriers to prevention, disclosure of disease status as well as to testing for thalassaemia [7]. Additionally, a number of studies worldwide showed that attitudes toward prenatal diagnosis were related to religious convictions. Muslim couples, for instance, have been reported to refuse prenatal diagnosis on religious grounds [10,11]. Studies on knowledge, attitudes and practices related to thalassaemia are relatively scarce in Malaysian context. Our study, the first nation-wide survey in Malaysia, aimed to determine differences in Malaysian public awareness, perceptions and attitudes toward thalassaemia and thalassaemia screening practices. In particular, the findings hope to provide insights into culturally congruent educational interventions to increase knowledge and cultivate positive attitudes toward prevention of thalassaemia in multi-ethnic communities.

## Methods

### Sample

Interviews were conducted between July and December 2009 using a computer-assisted telephone interview (CATI) system. The telephone numbers were generated randomly by the computer from the latest electronic residential telephone directory (2008) of all 13 states and 3 federal territories in Malaysia. The sample was stratified by states and territories to ensure geographic representation. The sample size required for an approximately 10 million of population aged 18 to 55, for an accuracy level of 0.95 with a confidence interval of ± 2.0% was 2400. For the estimate response of only 10% (due to invalid and inactive phone numbers, unreachable, refuse to participate), 24,000 numbers were randomly generated for inclusion in the study from over 2.6 million numbers registered in the 2008 telephone directory. To be eligible for telephone interview, participants had to be Malaysians, aged between 18 to 55 years old, residing in the contacted household, and have heard of thalassaemia. Only one person per household was surveyed. If more than one eligible person is found in a household, one person will be selected in a separate random drawing from among all eligible participants. Interviews were conducted between 5.30 pm and 10.00 pm on weekdays and from 12.00 p.m. to 7.00 p.m. on weekends or public holidays to avoid over-representation of unemployed participants. Unanswered calls were attempted at least two more times on separate days before being regarded as non-responses.

### Instrument

The questionnaire comprised 44 questions, divided into 4 parts (Appendix 1). Firstly, participants were asked if they have heard of thalassaemia or a disease with low numbers of red blood cells or shortage of blood. Only those who answered "yes" will proceed with the survey. Subsequently, they were asked from where they had obtained information about thalassaemia. Participants were also queried if they have any family members or blood relatives who are thalassaemia carriers or with thalassaemia major.

Participants' knowledge about thalassaemia was assessed across several domains: 1) general knowledge of thalassaemia (5-item); 2) knowledge of thalassaemia major (6-item); 3) knowledge of thalassaemia carrier (8-item); 4) knowledge of prevention of thalassaemia major (2-item). For each question, a correct response was given a score of one, and an incorrect or 'don't know' was scored as zero, for a total possible score of 0-21, with higher scores indicating better knowledge.

The second part assessed attitudes toward thalassaemia (4-item), where participants were asked for their views on premarital screening, marriage between individuals who are both carriers, pregnancy of carrier couples and termination of pregnancies affected with thalassaemia major. In the last section, married participants were asked about their practices regarding thalassaemia screening (8-item). Demographic questions (8-item) were asked after completion of the survey questions. The questionnaire was adapted and modified from previous published literature [7,10,12,13]. The questionnaire was translated into Bahasa Malaysia (the national language of Malaysia) and Chinese (Mandarin). The translated questionnaires were reviewed by secondary translators and back translation was conducted on the primary translated version. All back translations were reviewed by the researchers where edits to the target language version were made as necessary. The questionnaire was content validated by a panel of experts who are also researchers of the study to ensure that the items have acceptability content validity. After some minor modifications the questionnaire was reevaluated by the same panel of experts. The final draft version was pilot tested on 20 random samples of different ethnic population from the telephone directory. The questionnaire was face validity and tested on the Computer Assisted Telephone Interview (CATI) system.

A team of trained interviewers from different ethnic groups performed the interviews and each interviewer was assigned to interview respondents of a similar ethnic group. Informed consent was obtained verbally. Participation was voluntary and verbal consent was obtained before the start of the telephone interview. The study was approved by the Medical Ethics Committee, University Malaya Medical Center, Kuala Lumpur, Malaysia.

### Analyses

Data was analyzed using SPSS 17.0 for Windows. Values of *P *≤ 0.05 were considered significant. T-tests and one way analysis of variance (ANOVA) were used for comparisons of means; the chi-square test was used to test the significance of differences in percentages. Post hoc comparisons using the Tukey's HSD test were conducted to evaluate pairwise differences among the means. Cronbach's alpha was used to assess the internal consistency of the knowledge items in the study questionnaire, with values of at least 0.7 indicating acceptable internal consistency.

Calculation of crude odds ratio (OR) and comparisons of socio-demographic characteristics of participants that have and have not heard of thalassaemia were performed. Multiple regression analysis was used to determine socio-demographic predictors of knowledge score. Variables with a p-value < 0.2 were entered into the multiple linear regression model.

## Results

### Participant's characteristics

**Figure 1:**
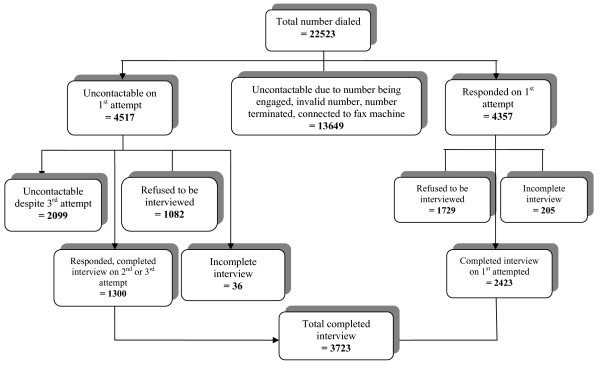
**Illustration of the CATI process of the survey**.

Figure 1 provides the flowchart of the CATI process. A total of 22523 call attempts were made, resulting in 3723 responding households. The response rate of 54.9% was computed as the number of completed interviews divided by the number of contacted households (6777). The Chinese have the highest non-response rate (56.5%), followed by the Indians (40.4%) and the Malays (39.1%). Table 1 shows socio-demographic characteristics of the survey participants. The mean age of the study participants was 35.0 (SD ± 9.9).

**Table 1:** Distribution of socio-demographic characteristics and proportion that have heard of thalassaemia (N = 3723)

Socio-demographic variables	All participants N = 3723 n (%)	Have heard of thalassaemia n (%)	
		
		Yes n = 2846	No n = 877
**Gender**			
Male	1202 (32.3)	809 (67.3)	393 (32.7)
Female	2521 (67.7)	2037 (80.8)	484 (19.2)
**Marital status**			
Never married	1213 (32.6)	970 (80.0)	243 (20.0)
Ever married	2510 (67.4)	1876 (74.7)	634 (25.3)
**Ethnicity**			
Malay bumiputra	2337 (62.8)	2050 (87.7)	287 (12.3)
Chinese	837 (22.5)	484 (57.8)	353 (42.2)
Indian	332 (8.9)	153 (46.1)	179 (53.9)
Non-Malay bumiputra	205 (5.5)	154 (75.1)	51 (24.9)
Others	12 (0.3)	5 (41.7)	7 (58.3)
**Age**			
18-35	895 (24.0)	658 (73.5)	237 (26.5)
26-35	984 (26.4)	798 (81.1)	186 (1839)
36-45	1132 (30.4)	907 (80.1)	225 (19.9)
46-55	712 (19.1)	483 (67.8)	229 (32.2)
**Highest education attainment**			
Primary school	275 (7.4)	139 (50.5)	136 (49.5)
Secondary school	1983 (53.3)	1431 (72.2)	552 (27.8)
University	1465 (39.3)	1276 (87.1)	189 (12.9)
**Occupation**			
Self employed	315 (8.5)	219 (69.5)	96 (30.5)
Professional and managerial	986 (26.5)	858 (87.0)	128 (13.0)
Skilled workers	610 (16.4)	492 (80.7)	118 (19.3)
Unskilled workers	288 (7.7)	172 (59.7)	116 (40.3)
Student	499 (12.1)	332 (73.9)	117 (26.1)
Housewife	833 (23.7)	659 (74.3)	227 (25.7)
Unemployed	122 (3.3)	82 (67.2)	40 (32.8)
Retired	70 (1.9)	35 (50.0)	35 (50.0)
**Average monthly household income (RM) **^† ^‡			
<2000	1535 (41.9)	1083 (70.6)	452 (29.4)
2001-4000	1249 (34.1)	984 (78.8)	265 (21.2)
>4001	876 (23.9)	753 (86.0)	123 (14.0)
**Locality**			
Urban	2393 (64.3)	1794 (75.0)	599 (25.0)
Rural	1330 (35.7)	1052 (79.1)	278 (20.9)

† Number of respondents less than 3723 (total respondent) due to non-response.

‡ The national average monthly household income in Malaysian Ringgit (RM) is RM3,686 (US$1 = RM3.24, as of April 2, 2010)

### Knowledge

Of the total contacted respondents, 76.4% (n = 2846) have heard of thalassaemia or a disease with low numbers of red blood cells or shortage of blood. Female, Malay ethnicity, married, middle-age, high education, professional and managerial occupation respondents were more likely to have heard of thalassaemia (Table 1). Relatively higher percentage of rural residents was aware of thalassaemia or a disease with shortage of blood. The three most frequently cited sources of information about thalassaemia were mass media (83.3%), family and friends (15.2%), and health care providers (8.9%). Of the 2846 participants that were aware of thalassaemia, 6.3% (n = 180) reported have had family members or blood relatives who are thalassaemia carriers or with thalassaemia major.

The majority of the participants knew that thalassaemia is a genetic disease (69.7%), and blood tests can be performed to determine if a person has thalassaemia (92.8%). Only 57.0% knew that an individual can be a thalassaemia carrier or be affected with a major form of the disorder. An even lower proportion (23.5%) was aware that a person can possess either alpha or beta thalassemia.

A large proportion surveyed (52.1%) were not aware that β-thalasaemia major patients require regular blood transfusions throughout life and there were misconceptions that thalassaemia major patients are mentally retarded (36.2%). Many misperceived that the life span of a thalassaemia carrier is short (62.4%) and a large majority of participants (79.7%) erroneously believed that a thalassaemia carrier may later develop thalassaemia major. When asked whether *Children born to either one parent who is a thalassaemia carrier will be at risk of having thalassaemia*, 77.5% provided the correct answer. However, when asked whether *Children born to either one parent who is a thalassaemia carrier will be at risk of having thalassaemia major*, and *Couples where both are thalassaemia carriers will certainly give birth to thalassaemia major children and will not conceive any healthy children*, the proportion with wrong answers were 74.8% and 69.0%, respectively. With regard to prevention, the majority were aware that the partner of a thalassaemia carrier should undergo blood tests (89.8%) and prenatal diagnosis is necessary for couples who are both thalassaemia carriers (84.4%).

The internal consistency (Cronbach's alpha) for the 21-item close-ended knowledge questions was 0.86. The mean total knowledge score for the overall sample was 11.85 (SD ± 4.03) out of a possible score of 21. Statistically significant differences (*P *< 0.05) were observed in the mean total knowledge score by age groups, education attainment, employment status, and average household income (Table 2). Multiple linear regression analysis shows that only average household income significantly predict knowledge score. The adjusted *R*^2 ^for this model was 0.007, which implies that the model explains 0.7% of the variability. The regression model was significant, F (9, 2836) = 3.158, *P *< 0.01 (Table 2).

**Table 2:** Socio-demographic differences in mean knowledge score and multiple linear regression analysis of socio-demographic variables predicting knowledge of thalassaemia (N = 2846)

Socio-demographic variables		Total knowledge (0-21 items scale)		Linear regression model F (9, 2836) = 3.158, *P *< 0.01, Adjusted *R^2 ^*= 0.007
	**N^†^**	**Mean ± SD**	***P***	**Unstandardized beta ** **coefficient (SE)**

**Gender**				
Male	809	11.80 ± 4.06		-
Female	2037	11.87 ± 4.03	0.71	
**Marital status**				
Never married	970	11.83 ± 4.05		-
Ever married	1876	11.86 ± 4.02	0.83	
**Ethnicity^‡^**				
Malay bumiputra	2050	11.90 ± 3.86		
Chinese	484	11.58 ± 4.63		
Indian	153	12.08 ± 4.19	0.56	-
Non-Malay bumiputra	154	11.88 ± 4.09		
Others	5	11.20 ± 6.03		
**Age**				
18-35	658	11.64 ± 4.04		-0.036 (0.20)
26-35	798	11.93 ± 3.97		0.13 (0.24)
36-45	907	12.03 ± 3.89	0.17	0.29 (0.23)
46-55	483	11.67 ± 4.38		Reference
**Highest education attainment**				
Primary school	139	11.39 ± 4.12		-0.30 (0.39)
Secondary school	1431	11.70 ± 3.97	0.023*	-0.15(0.18)
University	1276	12.07 ± 4.09		Reference
**Occupation**				
Employed	1741	12.01 ± 4.06		0.18 (0.17)
Unemployed	1105	11.60 ± 3.98	0.008**	Reference
**Average monthly household income (RM)**				
<2000	1083	11.47 ± 3.86		-0.68 (0.22) **
2001-4000	984	11.87 ± 4.05	0.000***	-0.39 (0.20) *
>4001	753	12.35 ± 4.23		Reference
**Locality**				
Urban	1794	11.96 ± 4.14		0.13 (0.16)
Rural	1052	11.66 ± 3.85	0.052	Reference

* *P *< 0.05; ** *P *< 0.01; *** *P *< 0.001

^† ^Subtotals may vary owing to missing data

### Attitudes

As shown in Table 3, the majority of the participants viewed that premarital screening for thalassaemia is necessary for the general public (90.6%). Only 34.7% were of the opinion that couples who are thalassaemia carriers should not marry. There were significant differences of opinion as to whether couples who are thalassaemia carrier should have children. The proportions who agreed that thalassaemia carrier couples should not have children were significantly lower in Malay participants compared with other ethnic groups. Spearman rank correlation analysis showed a weak but significant positive correlation between agreement in opinion that couples who are thalassaemia carriers should not have children and educational level (*r *= 0.058, *P *< 0.001). Likewise, significantly lower agreement among the Malay participants was found for the question regarding termination of an affected pregnancy with thalassaemia major. Similarly, Spearman rank correlation analysis showed a weak but significant positive correlation between agreement in opinion of termination of pregnancy and educational level (*r *= 0.079, *P *< 0.001).

**Table 3:** Comparison of attitudes toward thalassaemia among ethnic groups

Items	Proportion of agreement (%)					
	
		Ethnic group				
		
	All participants (n = 2846)	Malay bumiputra (n = 2050)	Non-Malay bumiputra (n = 154)	Chinese (n = 484)	Indian (n = 153)	*P*
Premarital screening for thalassaemia is necessary for the general public	90.6	91.4	87.0	88.4	91.5	NS
Couples who are thalassaemia carriers should not marry	34.7	34.9	35.1	33.7	34.0	NS
Couples who are thalassaemia carriers should not have children	31.3	27.1	29.2	48.3	34.0	*P *< 0.001
Termination of a pregnancy with thalassaemia major is necessary as it not only brings suffering to the affected child, but it is also a burden to the family, community and country	36.6	36.0	25.3	41.5	39.2	*P *< 0.01

### Practices

When unmarried participants were asked if they had undergone thalassaemia screening, only 13.6% (of total 966 responses) reported that they had been screened for thalassaemia. Among these, the majority were Chinese (30.8%), followed by Indians (17.4%), Malay bumiputras (8.6%), and non-Malay bumiputras (8.2%) (*P *< 0.001). A majority (86.9%, n = 726) of unmarried participants who had not been screened indicated willingness to undergo thalassaemia screening. Among reasons for the remaining 13.1% (n = 109) who indicated unwillingness to undergo thalassaemia screening were - perceived not to be at risk (46.8%), afraid of test result (20.2%), afraid of being discriminated (15.8%), no knowledge of thalassamia testing (8.3%), no idea where to do the test (3.7%), and others (5.2%). There were no significant differences in having screened for thalassaemia by educational level, income and locality. The prevalence of thalassaemia carrier rate was 6.9% (n = 6) among the 131 participants who reported having been screened for thalassaemia.

Of a total of 966 responses, 22.7% (n = 219) indicated they will not continue to be with their partners if both are thalassaemia carriers. Among the reasons were - they do not want children with thalassaemia major (85.4%), family objections (4.5%), predestination of God's will (4.0%), empathy (3.6%), and others (2.5%). A total of 68.2% (n = 659) indicated unwillingness to abort their baby if the child has been diagnosed with thalassaemia major. The main reasons were predestination of God's will (30.8%), religious prohibition (30.7%), empathy (18.6%), rights for babies to live (14.6%), hope for availability of new treatment and cure for thalassaemia (3.2%), and others (2.1%).

## Discussion

Although a majority of respondents have heard of thalassaemia in Malaysia, a substantial percentage still remains unaware. Awareness of thalassaemia was higher in the high income, high education, and professional and managerial categories. This result indicates that education programmes to increase awareness of thalssaemia should be concentrated more in the low-acculturated groups. Disparities across the three main ethnic groups (Malay, Chinese, and Indian) in awareness of thalassaemia are not known and this warrants further investigation. Educational efforts are needed to raise awareness, particularly among the Indian groups, to bridge the awareness gaps between people of different ethnic backgrounds in Malaysia. It has been suggested that community-based education program work best to address the knowledge disparities in multiethnic country [12].

The mean knowledge score of 11.85 out of a possible score of 21 may reflect a general lack of knowledge among the study participants. Of particular important was the misconception that carriers of thalassaemia will manifest the disease. It is utmost important to dispel this misconception so that carriers of this genetic disorder are not stigmatized by society. Participants' responses also indicated that specific knowledge regarding the existence of different types of thalassaemias (α- and β-thalassaemia), the genetic nature of the disease, and its pattern of inheritance were poor. These knowledge deficits may result in unnecessary anxiety among the public and may have profound emotional effects on the carriers of thalassaemia. Public health messages should focus on disseminating information about the differences between the various types of thalassemia and that the disorder only manifests severely in individuals who are homozygotes. It has been reported that community health education and outreach programmes have helped in controlling the prevalence of the disease and greatly reduced its health impacts [14-16]. Owing to the genetic complexity of the disorder, it remains a challenge to educate the low-acculturated communities [7]. It has been suggested that effective communication can be established with audio-visual aids and personal experience sharing [6].

The majority of participants in this study expressed very positive attitudes toward screening for thalassaemia. Therefore, efforts to promote screening are likely to receive favourable responses from the Malaysian general public. Facilities for premarital and prenatal diagnosis to confirm the molecular mutations involved, and genetic counseling services will contribute to a reduction in the numbers of babies born with thalassaemia major [1,8,9]. Of concern is a considerable minority of participants that perceived premarital screening as unnecessary for the general public. The current quantitative study limits in-depth exploration of the reasons for refusal. The reasons participants perceived premarital screening as unnecessary warrant further investigation in future qualitative studies. Literature reports have indicated that when extended family members were approached to identify carriers in the family tree, the responses were usually unfavourable due to the fear of being stigmatized, in particular, many concerned carrier status may tarnish reputation and affect future marriage prospects [7,17]. Religion is also believed to have a significant impact on decisions about screening [10].

In general, termination of pregnancy is not a consideration among the Asians because of a complex web of moral, cultural and traditional religious values of the family and community. Under the section 312 of the Penal Code (Amendment) Act 1989 of Malaysia, an abortion is only permitted if the pregnancy is likely to result in danger to the mother's physical and mental health. Unfortunately there are very few studies looking at societal perspective on the acceptability of termination of pregnancy in Malaysia context. Several studies showed religious beliefs to be associated with refusal for prenatal diagnosis and termination of affected foetuses among high risk couples [10,18,19]. In this study, only slightly more than one-third of our study participants supported selective termination of affected foetuses. In addition, a majority of the married participants were unsupportive of selective termination of foetuses diagnosed with thalassaemia major. As such, carrier screening at an early age (in school) and premarital screening programs aimed at identifying individuals before marriages may be more effective and culturally acceptable among our communities compared to prenatal diagnosis carried out in the antenatal clinic. Implementation of mandatory national premarital screening program, and screening young and unmarried women for detection of carriers have dramatically reduced the incidence of infants born with major thalassaemia in several countries worldwide [20]. For example, premarital screening to identify carrier couples and subsequently provision of counseling in Iran has resulted in a 70% reduction in the annual birth rate of affected infants and a large amount of medical expenses [21,22]. It is of interest to note that there are ethnic differences regarding termination of fetuses affected by thalassaemia major in this study. On the whole, the proportions of participants who support termination of fetuses were lower among the Malays compared to the Chinese and Indians. This is associated with perceived cultural and religious restrictions on abortion among the Muslim [23]. In this context, premarital screening program coupled with antenatal diagnosis and legalization of abortion before the 16 weeks of gestational age are recommended [22]. In short, given the diversity in attitudes toward termination of fetuses within different religions, thalassaemia prevention programmes should consider the beliefs and preferences of individuals in multiethnic society [10].

The most worrisome finding in this study is that the proportion of married participants screened for thalassaemia constituted approximately only one-eight of all the married participants interviewed. As a lack of perceived risk was the main reason for those unwilling to be screened, the general public should be educated that there is a fairly high prevalence of thalassaemia in Malaysia. Health care providers are encouraged to discuss thalassaemia as a public health problem in Malaysia and enhance public awareness. As perceived severity of thalassaemia was found as the major influence in a women's decision about prenatal diagnosis [24], physician-patient communication should emphasize on severity of the disorder and its treatment and the importance of screening for thalassaemia. An issue of considerable concern was a small proportion of participants refused to be tested for thalassaemia due to the fear of being diagnosed, and subsequently being discriminated and stigmatized. Thus, thalassaemia awareness and screening programs must be specifically and carefully designed to promote screening participation yet prevent prejudice and discrimination against carriers. It is essential that genetic counseling for thalassaemia to be provided alongside with confirmation of the disorder so that patients are reassured of their options and are able to make inform decisions.

It was reported that the majority of carriers identified in a high-school screening program remembered their status, favourable of knowing the status of their partners, and willing to take options for reproductive counseling and prenatal diagnosis [25]. Likewise in this study, among the young unmarried participants, near 23% indicated not willing to continue to be with their partners if both are thalassaemia carrier, and 32% indicated willingness to abort their baby if the child has been diagnosed with thalassaemia major. An earlier study in Montreal showed that high-schools students have high level of interest in thalassaemia screening, with participation rate of near 80% [26]. This again indicates that school-based carrier screening may lead to favorable outcomes.

This study has several limitations. First limitation of this study is that all data were collected via self-report; therefore, reporting bias due to socially desirable attitudes and behaviors might exist. Further, attitudes do not necessarily predict actual behaviors [27]. In addition, all data were collected via telephone interview and subject to the limitations of any telephone interview survey namely telephone sampling bias and reporting bias. The telephone interview study only included households with fixed-line telephones, therefore, those with mobile telephones only and household without fixed-line telephones (who are proportionately more prevalent among socio-economically disadvantaged groups) were under-represented [28]. Despite these limitations, this study constitutes a large sample size and samples were selected as being representative of the general population. The proportion of ethnic groups, highest educational achievement, and average household income of the sample matches the proportion in the general population [29], suggesting that these results may be reflective of national trends. Another advantage is that the high response rate of 54.9% was achieved, with 3723 completed survey over 6777 contacted household, compared to self-administered survey [30]. The study is also unique because the sample is multi-ethnic, encompassing the three main ethnic groups (Malays, Chinese, and Indians) in Southeast Asia, which has not been reported elsewhere, thus offers many insights that have practical relevance to other Southeast Asia countries. Lastly, reliability of the knowledge scale was high (Cronbach's alpha: 0.86).

## Conclusion

The Ministry of Health, Malaysia has announced an intention to start a national screening programme for thalassaemia. This study has identified key areas which need to be highlighted and emphasized in any public education and awareness campaigns for thalassaemia screening in Malaysia. Data from this nation-wide survey has specifically pointed out knowledge deficits regarding the genetics and pattern of inheritance of thalassaemia, thus, an integral part of any public educational intervention should be information on the molecular basis of thalassaemia. Secondly, the results reflect socio-demographic differences in awareness of thalassaemia, as such, a tailored campaign is necessary to target the less knowledgeable groups. Thirdly, the social stigma associated with the diagnosis of thalassaemia needs to be addressed in order to increase motivation for the general public to be screened for thalasseamia.

This study has also highlighted a number of important implications for service provision in Malaysia. The study found that socio-cultural factors had an important influence on perceptions towards selective termination of foetuses with thalassaemia major. This therefore suggests that when thalassaemia prevention is offered to the population through carrier screening, premarital or prenatal diagnosis, socio-economic, cultural and religious factors must be carefully taken into account. Understanding these factors will provide essential insights into successful strategies to reduce births of thalassemia major children. This study clearly shows that reducing the frequency of thalassaemia is feasible and acceptable in a country with very diverse demographic and cultural beliefs such as Malaysia.

## Competing interests

The authors declare that they have no competing interests.

## Authors' contributions

LPW participated in formulation of the research questions, conception and design of study, data collection, analysis, interpretation and writing the manuscript. EG and JMAT participated in formulation of research questions, critically revision of the manuscript. All authors read and approved the final manuscript.

## Pre-publication history

The pre-publication history for this paper can be accessed here:

http://www.biomedcentral.com/1471-2458/11/193/prepub
